# Radical Cure for Hernia

**Published:** 1835

**Authors:** 


### Art. II.—Radical Cure of Hernia.

We have more than once taken a favorable notice of the invention by Mr. Stagner, of Kentucky, of a wooden headed truss, for the *radical* cure of Hernia. Mr. S. is not a professional man, but his suggestion soon attracted the favorable notice of the medical gentlemen of the region in which he resided, and among the rest of Dr. Hood, a graduate, we believe, of Transylvania University; who visited the eastern cities and sought to attract to the invention, the notice both of the people and the profession, and considerable success attended his efforts. It was soon discovered, however, that the form of Stagner's wooden pad (which was invariable) did not answer for every case; and this led to efforts, by different surgeons, to effect such modifications of figure, as might render it of general applicability. Among those who have exerted themselves in this laudable object, are Dr. Hood, whom we have just named, and Mr. Chase, of Philadelphia; each of whom has produced modifications of size and form, calculated, or at least intended, to augment its advantages. The deep interest of the subject, induced the Philadelphia Medical Society, to appoint a committee, "To investigate the character of Stagner's Truss, and other proposed means of radical cure in Hernia," and on the 5th and 12th of December, 1835, the committee, consisting of Drs. Reynell Coates, William Ashmead, and Isaac Parrish made their report, which was published in the American Journal, and from which we propose to give a few extracts.

After the citation of a number of cases, going to show the efficacy of the modifications of Stagner's block in restraining and curing Hernia, the report presents us with the following testimony as to the former point:

"The committee are decided in the opinion, that the retentive power of solid blocks exceeds, *cæteris paribus*, by considerable difference, that of pads composed of softer materials. If there could be any exception taken to this rule, it would be in favor of pads formed of very firm but highly elastic materials; but the only substance of the latter character, now employed in the construction of truss-pads, is the gum elastic, and against the direct application of caoutchouc to the skin, there are strong physiological objections. Moreover, the committee is by no means prepared to advocate the superiority of elastic pads, in the present state of their knowledge. Whatever they may gain in facility of application, they lose in certainty of action. The great excellence of the solid truss-block is its perfect precision, and if required to adapt itself to changes of position in the part to which it is applied, it can be enabled so to do by the elasticity of the spring of the truss.

Two circumstances should be stated in this place;—the incompressibility of very firm pads and hard blocks, renders it of the utmost importance that their form should be accurately adapted to the particular parts on which they are designed to act, and that they should be carefully placed and secured in correct relation with those parts. Carelessness in either of these respects would incur dangers, grave in just proportion to the power and usefulness of the apparatus. Hence considerable anatomical and surgical skill are requisite, both in the contrivance and the application of trusses armed with such pads or blocks, and they can never be permitted to pass, with safety, out of the hands of surgical scholars and practitioners. Again, the application of such machines, in early infancy, is deemed by the committee both unnecessary and improper.

With regard to other dangers and inconveniences, your committee will merely remark, that the charge of danger of general peritoneal inflammation from the communicated irritation of the blocks, when their application is directed by competent skill, is deemed nugatory, and in opposition to both well-known pathological laws and the direct evidence of experience. The danger of suffering from the production of external inflammation is, in general, slight and unworthy of much thought, and when epidemic tendencies or peculiar states of constitution render the patient liable to diffuse inflammation of the cellular tissue, the danger from the irritation of the blocks, even when employed with force, in order to carry out the theoretical indication mentioned in the commencement of this report, is certainly not greater than that produced by very trivial and unavoidable accidents, such as slight scratches and abrasions on other parts. But even the correctness of the doctrine which attributes the radical cure to extensive adhesions and callosities, is of very doubtful character, and the necessity of employing the blocks in such a manner as to produce considerable irritation, is by no means decided. The committee, therefore, think that these alleged dangers do not appreciably affect the permanent retentive power of the apparatus.

There is apparently one slight additional security against the descent of the bowel in inguinal and certain other hernia, which is often consecutive upon the use of the hard block for a moderate length of time. This is the rapid absorption of the deposits in the subcutaneous cellular tissue, and sometimes in the dermoid tissue also, which permits the block to act almost immediately upon the tendinous canal, thus effectually closing the neck of the hernial sac, of which it very probably produces the obliteration. It is possible that the temporary security produced by this means, which very slightly opposes the formation of a new sac, has been a cause of deception with regard to certain cases reported as radical cures, but which have been sub,ected to relapse. Several cases of this character have been before the committee, but they deem it inexpedient to dwell upon the subject farther than to caution the profession generally against hasty conclusions on medical and surgical questions.

In regard to the retentive power of particular blocks, the committee are prepared to express their warm approval of the inguinal block of Mr. Chase, in which, at present, it can suggest no improvement. It also approves of Dr. Hood's ventro-inguinal block, with the pa:abolic projection, but considers a more perfect instrument, to fulfil the same purposes, both possible and desirable. The same sentence is pronounced upon the femoral block of Mr. Chase; but in the latter case some very important amendments have been made, which appear to meet the views of the committee, and will be verbally commented upon at the close of this report. Their practical application will be made the subject of future investigation.

The committee feel some regret in condemning the mechanical principles upon which the concave ventro-inguinal, the femoral, and the more complex umbilical blocks of Dr. Hood, are constructed, the action of the second of these instruments having been proved inefficient in certain particular cases within the knowledge of the Society generally. The evidence with regard to the action of the common inguinal blocks of Mr. Stagner and Dr. Hood, has been already offered, and of course they fall under the objection of uncertainty of retention. The committee do not deem it necessary to be more explicit in commenting either upon these blocks or upon the drawers, belts, or other apparatus mentioned in the specification of Dr. Hood. They hold themselves prepared to meet, if necessary, any remarks upon these questions during the subsequent debate. With regard to their opinion of the other umbilical blocks, a judgment has been expressed already."

On the very important question of a *radical* cure, from the use of the wooden block, the report holds the following language:

"With respect to the question of the radical cure of hernia, by means of the best of the instruments which have passed in review, it should be borne in mind, that success in cases of umbilical hernia in young children, is almost general, when methodical bandaging has been judiciously employed. That in other varieties of hernia, affecting subjects of similar ages, success is by no means rare, under the operation of trusses with soft pads; that in children over ten years of age, it becomes rather uncommon; that in youths between the age of puberty and that of twenty years, it becomes rare; and after the latter period, *very rare*. These remarks premised, the committee are of opinion, that the chances of radical cure depend mainly upon the retentive power of the apparatus employed—and their opinion on this subject has been already expressed. It would be wrong to enter upon the calculation of probabilities, without much more extended observation than has yet been possible, but the committee have no hesitation in stating that the action of the several blocks recommended in the last section appears to offer much more prospect of radical cure, even under unfavorable circumstances, than any apparatus previously offered to the public, and which has fallen under their notice after considerable research."

As to the *modus operandi* of the block, in effecting a radical cure, the committee incline to the opinion, that an accurate, perfect, and constant retention, of the protruding part, is far more important than the excitation of inflammatory action. We shall not follow them in their reasonings on this subject, which embrace a reference to the spontaneous closure of the *tunica vaginalis testis*, in childhood; for no certainty will attend our knowledge on this subject, until accumulated dissections shall have been made of those who have been radically cured; and of course this will require the lapse of many years.

In the conclusion of their report, the committee express themselves on the authorship of these inventions in the following language:

"The question of novelty is one in which the committee feel but little interest, being convinced that that of utility is of vastly more importance, but it will merely remark, in passing, that the almost endless variety of forms given to truss pads from time immemorial, and the generality of the directions for forming them, as given by the highest surgical authority, would make it a task as vain as useless to render *suum cuique* in this department of surgery. The blocks of Mr. Chaise, are, however, mathematical figures, capable of accurate expression, and, if it be thought necessary, the committee will endeavor, under the injunction of the society, to investigate his individual claims. In most of the other cases, they regard the inquiry as nearly hopeless.

If originality on the score of the material used in the construction of the blocks be claimed, the committee can only state its inability to recall any instance of the use of wood as a pad in hernia, occuring under the observation of its members prior to Mr. Stagner's invention, but it has been long familiar with the use of ivory."

We shall terminate this notice, by referring the reader to the cover of this number, for the advertisement of Dr. Crittenden, who is sojourning in Cincinnati, as the agent of Mr. Chase, and will undertake to apply wooden headed trusses in all cases that may be presented to him.

